# Perspectives on research in hypertension

**Published:** 2009-02

**Authors:** YK Seedat

**Affiliations:** Nelson Mandela School of Medicine, University of KwaZulu-Natal, Durban

## Abstract

**Summary:**

This is a review of my published research on hypertension over 45 years on the three main racial groups residing in KwaZulu-Natal and its main city Durban. These three groups are blacks – mainly Zulu, whites and Indians. The research focused mainly on epidemiology, determinants of the aetiology of hypertension, clinical features, varying responses to hypotensive agents among the racial groups, complications that result from hypertension and the control of hypertension.

## Hypertension in blacks

Hypertension is a major disease in the black population of sub-Saharan Africa.^1^-^4^ Our age-corrected prevalence studies showed that in the adult black population of Durban, hypertension [World Health Organisation (WHO) criteria] was highest in adult urban Zulus (25%), intermediate in whites (17.2%) and lowest in the Indian population (14.2%).^5^-^10^ The mean systolic blood pressure (SBP) and diastolic blood pressure (DBP) in urban Zulus was lower than in West Indians,^11^ Nigerians^12^ and African-Americans.^13^

The difference in mean arterial pressure between the African-American and the urban Zulu population is probably due to the African-American being acculturated for 300 years, whereas the urban Zulu have been acculturated since only the turn of the last century. It is possible that differences in the years of acculturation of African-Americans, West Indians, Nigerians and urban Zulus explain the differences in mean SBPs and DBPs.^5^

Hypertension occurs at an earlier age in African-Americans than in whites of the USA.^13^ We have found that females between the ages of 35 and 40 years have a higher prevalence than males.^5^,^8^,^9^ The higher prevalence of hypertension in our urban blacks, particularly in females under 40 years, probably explains the younger age groups of hypertensive patients at autopsy.^14^

In a study of 4 993 rural Zulus, the overall prevalence of essential hypertension was 8.37% (8.78% females, males 7.4%).^15^ The mean arterial pressure in relation to age and gender was not as high as in urban Zulus. A diastolic blood pressure of ≥ 95 mmHg was present in 45% of the subjects and 1.46% had a diastolic blood pressure of ≥ 110 mmHg. This study suggests that hypertension is not a major problem in rural Zulus and that large case-finding and intervention programmes should be directed to the urban black population of South Africa in situations where resources are limited.

## Determinant factors of hypertension in blacks

A study was carried out to evaluate the relationship between blood pressure, plasma renin activity, serum aldosterone and patterns of urinary sodium and potassium, and potassium excretion rates in urban Zulus, rural Zulus and Indians, in order to explain the high prevalence of hypertension in the urban adult Zulu (25%) versus the rural Zulu (8.3%). Urinary sodium and potassium levels were not significantly different between urban and rural Zulus. There was no association between levels of sodium excretion and blood pressure. Urinary potassium levels correlated negatively with blood pressure in rural Zulus and Indians but not in urban Zulus. The sodium:potassium ratio was significantly lower in rural Zulus than in urban Zulus. The sodium:potassium ratio in Indians was not significantly different from that of Zulus. There were overt differences in potassium transport, suggesting that blacks have a more limited capacity to exchange intracellular sodium for intracellular potassium and a higher percentage of this exchange is dependent upon oubain-sensitive mechanisms.^16^ We have found that lymphocyte intracellular level was significantly higher in hypertensive Zulus compared to normotensive Zulus.^16^,^17^ The kallikrein excretion levels in the urban Zulus was the same as our Indian subjects.^18^,^19^

We have found a higher plasma renin activity (PRA) in rural Zulus compared to urban Zulus. This suggests that the cause of a low PRA in blacks may not be genetic. However, there was no correlation between PRA and urinary sodium, suggesting that dietary sodium was not a factor. There was no correlation between renin and aldosterone in hypertensive urban Zulus, suggesting a defect in the renin-aldosterone system in the patients.^16^

Obesity is an important cause of hypertension, especially in urban compared to rural Zulu females.^5^,^8^

We have found important social factors that predispose to hypertension in our urban Zulu population. Hypertension is more common in the lower socio-economic status, in married people with several children, and those who spend hours travelling to work and live in deprived and less favourable townships. Urban hypertensive Zulus have a higher incidence of insomnia, anxiety, cigarette smoking, alcohol consumption, poor working conditions, lower educational status, greater numbers of children not working, lack of recreation or sports, and overcrowding in homes.^20^

An assessment of specific coping styles in rural and urban blacks was done to evaluate its contribution as cardiometabolic risk factor. In total, 608 apparently healthy blacks were included in a cross-sectional comparative study from the North West province in South Africa. The adapted and translated COPE questionnaire classified participants according to response, into active (AC) or passive (PC) copers.

Fasting resting metabolic syndrome (MS) indicators using the WHO definition (glucose, high-density lipoprotein and triglyceride levels, waist/hip ratio and hypertension prevalence) and associated MS values, i.e. fibrinogen, were obtained. Co-variates for all statistical analyses included age, body mass index (BMI) and lifestyle factors (alcohol consumption, smoking habits and physical activity). The only MS values prevalent in urbanised participants were higher prevalence rates of hypertension and higher fibrinogen levels (female only) compared to their rural counterparts.

Adding coping lifestyles, it was mainly the urbanised AC participants who indicated higher MS values (hypertension prevalence, glucose and fibrinogen levels) when compared to their rural and PC counterparts. Coping as a cardiometabolic risk was accentuated in the urbanised AC group, especially the men. The urbanised AC group with their higher blood pressure values and more MS indicators appear to have behaviorally an AC style but physiologically a dissociated AC style.^21^

## Clinical characteristics of hypertension in blacks

Hypertension in the hospitalised population pursues a rapid course, with death occurring frequently from cerebral haemorrhage, uraemia or congestive heart failure.^22^ Accelerated malignant hypertension in our hospitalised black population was 7%. The high incidence of accelerated malignant hypertension is probably due to the large numbers of untreated hypertensive patients who attend hospital at a late stage. Most of the patients in our study suffering from accelerated malignant hypertension had raised blood urea and this was usually the cause of death.^23^

A study titled ‘Risk factors and coronary heart disease in Durban blacks – the missing link’^24^ showed that the prevalence of coronary heart disease (CHD) in patients attending a dental clinic at a hospital was 2.4%. The percentage prevalence of selected risk factors were: hypertension (SBP ≥ 160 mmHg and/or DBP ≥ 90 mmHg) 28% (31.9% for males, 25.4% for females); protective levels of high-density lipoprotein cholesterol ≥ 20%, 81.3%; diabetes mellitus, 4.9% for males, 2.9% for females; smoking ≥ 10 cigarettes per day, 28.1% for males, 3.4% for females; obesity 3.7% for males, 22.6% for females. We found the Minnesota coding system for electrocardiographic changes of CHD and the Rose questionnaire to be unreliable for eliciting CHD in blacks. Hypercholesterolaemia was less common, and this may explain the low incidence of CHD in blacks.^24^

A study of the frequency of cardiac changes in 1 000 patients (500 blacks and 500 Indians) was undertaken over a period of seven years. Although congestive heart failure due to hypertension occurred in 16% of blacks, ischaemic heart disease did not occur. The rarity of myocardial infarction in blacks has been recorded in black hypertensive patients in sub-Saharan Africa.^25^ Hypertension is not uncommonly associated with cerebral atheroma and atheroma of the aorta, but the absence of coronary artery disease in the black hypertensive patient remains an enigma. 26 Diabetes mellitus occurred in 6% of the black patients.^22^

A 10-year post-mortem study of hypertensive patients (1965–1974) among 434 black patients at King Edward VIII Hospital, Durban showed that 41.9% had cerebrovascular complications, 25% had renal complications, 32.9% had cardiovascular complications, while only 10 patients had myocardial infarction.^22^ The average age of the patients suffering from hypertension at autopsy was 43 years (range 7–89), while the average age of the hypertensive patients with myocardial infarction was 54 years. Intracranial haemorrhage was responsible for 212 of the 223 (95%) patients suffering from cerebrovascular accident, and cerebral thrombosis constituted only 11 cases (5% ).^22^,^26^

Epidemiological studies showed that 90% of urban black hypertensive patients had hypertension that was undiagnosed, untreated or inadequately treated.^5^,^8^,^9^ Therefore it is highly desirable to have more effective programmes for the detection of hypertension in blacks. Equally desirable is an effective therapeutic compliance programme, since the follow-up of black patients suffering hypertension in detection and treatment trials for hypertension is poor. However, there are social, cultural, economic and political problems that make it difficult to apply the results of hypertension detection and treatment trials as practiced in the First World.

## Treatment of hypertension in blacks

Black hypertensive patients respond well to thiazide diuretics, vasodilators such as prazosin or labetolol, or reserpine.^27^ They respond less well to β-blockers such as propanolol^28^ or atenolol alone,^29^ or angiotensin converting enzyme inhibitors (ACE inhibitors) such as lisinopril^30^ or captopril,^31^-^32^ compared to Indian or white patients. However, the response is the same when β-blockers or ACE inhibitors are combined with a diuretic.^29^,^30^

There are varying responses to hypotensive agents in different racial groups – black versus white differences. The better hypotensive response in black hypertensive patients is probably due to the fact that, in comparison with whites, more blacks have an expanded intracellular volume and low plasma renin activity.^27^ In developing countries, where the majority of black people reside, the cost of drug therapy is important. Because of their low cost, thiazide diuretics are important baseline drugs in the treatment of hypertension.^33^-^34^

We have published our experiences of drug trials in black and Indian patients over the past 40 years. They include thiazide diuretics, indapamide^35^ clopamide, amiloride combined with hydrochlorothiazide, 36 reserpine with hydrochlorothiazide versus sotalol and hydroclothiazide,^37^ debrisoquine^38^ guanfacine,^39^-^42^ prindolol, 43-44 guanethidine,^45^-^46^ penbutolol,^47^ clonidine,^48^-^50^ methyldopa,^51^ prazosin,^52^-^54^ nisoldipine^55^ ketanserin,^56^ enhydralazine^57^ oxprenolol, 58 captopril,^59^ lisinopril,^60^ minoxidil^61^-^62^ and guanacline.^63^-^64^

## Hypertension in whites

In a random house-to-house study of 1 000 whites, the prevalence of essential hypertension according to WHO criteria was 22.76% (25.8% of males, 20.5% of females). This was lower than the prevalence in our study of urban Zulus and higher than that of urban Indians. The study revealed that the prevalence of hypertension in South African whites was higher than that in whites in the USA. The prevalence in males younger than 40 years was twice that in females of the same age. The high prevalence of diastolic blood pressures of 105 mmHg or more, together with the high prevalence of hypertension in white male subjects under 40 years of age could be an important factor in the aetiology of ischaemic heart disease in white male subjects in the 25- to 34-year age group.^7^

Coronary heart disease is the leading cause of death among the white and Indian populations of Durban. We did a community-based study of the white population of Durban, which is predominantly English speaking. A history of CHD was present in 9.3% of the subjects. The important risk factors were hypercholesteraemia, hypertension and smoking. The minor risk factors were obesity, hypertriglyceridaemia, a sedentary occupation and a history of CHD in the immediate family. Electrocardiographic abnormalities denoting CHD were present in 17% of the subjects.

A study of the major risk factors showed that 35.1% (age and gender adjusted) of patients had at least one major risk factor at the higher level (level A) and 33.8% (age and gender adjusted) at the lower risk levels (level B). When the combination of risk factors was taken into account, 15.2% and 28% had two major risk factors, one each at levels A and B, respectively. On average, the percentage of men and women with one risk factor or more increased with age. A protective high-density lipoprotein cholesterol ≥ 20% was present in 53.5% of the respondents.^65^

## Hypertension in Indians

In a random house-to-house study of 1 000 Indians, the prevalence of essential hypertension according to the WHO was 19% (females 22%, males 15%). This study showed that the prevalence was higher than in published data from India. Prevalence was lower than in our urban Zulu study. Blood pressure rose with age, but there was a greater rise in systolic than in diastolic hypertension. There was an association between hypertension and diabetes.^6^

Coronary heart disease is a major problem in migrant Indians throughout the world. In South Africa it has reached epidemic proportions. A community study was conducted in the metropolitan area of Durban to determine the prevalence and risk factors of CHD. A history of CHD was obtained in 15.3% (gender and age adjusted, 13.4%). The important risk factors in men were hypercholesterolaemia, hypertriglyceridaemia, diabetes and smoking, and in women diabetes, hypercholesteraemia and hypertriglyceridaemia. The minor risk factors were hyperuricaemia, sedentary occupation, obesity in women and a positive history of CHD.

A study of the major risk factors leading to CHD showed that 52% (gender and age adjusted, 45.5%) had at least one major risk factor at the higher level (level A) and 68% (gender and age adjusted, 61.9%) at the lower (level B) risk levels. Diabetes was strongly associated with a positive history of CHD. In 47.6% (gender and age adjusted, 48.2%) of the total group, resting ECG abnormalities were found that could be coded.^66^ We found that certain factors were significantly associated with hypertension, i.e. hypertriglyceridaemia, obesity, hyperuricaemia, hypercholesterolaemia, excess alcohol intake and diabetes. The association between hypertension, hypertriglyceridaemia, obesity, hypercholesterolaemia and diabetes suggests an underlying state of insulin resistance that could lead to coronary heart disease in the Indian population.^67^

We studied the blood pressure profiles in 154 medical students, aged 21, of whom 83 were Indians, 71 were black, 87 were male and 67 were female. We found that young black people had higher blood pressure readings than young Indian participants in the absence of metabolic abnormalities and had greater cardiac involvement. Borderline hypertension was not innocuous. Metabolic risk factors for CHD in Indian students were apparent at an early age. This study emphasises the need for prevention of risk factors leading to CHD at an early age.^68^

A cross-sectional dietary study as part of a CHD survey showed that the Indian subjects in our study had a low energy intake. Intake of dietary polyunsaturated (P) to saturated fat ratios (S) were high and the effect of such a high P:S ratio on the oxidation of low-density lipoprotein, with a high prevalence of CHD should be investigated as a possible risk factor.^69^

## Conclusion

The chronic disease hypertension and acquired immunodeficiency disease (AIDS) are major problems with regard to their control, management and treatment in southern Africa. The statement from the International Society of Hypertension is pertinent and relevant, that ‘failure of primary-care systems, together with a myopic view of disease targets among those who set international disease priorities has contributed to the staggering inequality in access to blood pressure-lowering treatments’.^70^
